# Comparison of oral iron chelators in the management of transfusion-dependent β-thalassemia major based on serum ferritin and liver enzymes

**DOI:** 10.12688/f1000research.128810.1

**Published:** 2023-02-10

**Authors:** Sulaiman Yusuf, Heru Noviat Herdata, Eka Destianti Edward, Khairunnisa Khairunnisa

**Affiliations:** 1 Division of Gastroenterohepatology, Department of Pediatrics, School of Medicine, Universitas Syiah Kuala - Dr. Zainoel Abidin Hospital, Banda Aceh, 23111, Indonesia; 2 Division of Hematology-Oncology, Department of Pediatrics, School of Medicine, Universitas Syiah Kuala - Dr. Zainoel Abidin Hospital, Banda Aceh, 32111, Indonesia; 3 Department of Pediatrics, School of Medicine, Universitas Syiah Kuala - Dr. Zainoel Abidin Hospital, Banda Aceh, 23111, Indonesia

**Keywords:** Alanine transaminase, aspartate transaminase, iron overload, deferiprone, deferasirox

## Abstract

**Background**: Excess iron deriving from a chronic transfusion and dietary intake increases the risk for cardiac complications in β-thalassemia major patients. Deferiprone and deferasirox are commonly prescribed to thalassemic patients who are at risk of iron overload. This study aimed to compare the performance and toxicity of deferiprone and deferasirox in β-thalassemia major patients.

**Methods**: A cross-sectional observation was performed on 102 patients with β-thalassemia major. Serum ferritin along with total, indirect, and direct bilirubin levels were measured. Levels of liver enzymes, transaminase (ALT), and aspartate transaminase (AST), were also determined. Ferritin correlations with serum ALT, AST, and total bilirubin were constructed based on Spearman’s rank correlation. Statistical differences based on the serum parameters were analyzed between deferiprone and deferasirox groups. The differences of iron chelators’ effects between those receiving short-term (≤7 years) and long-term (>7 years) blood transfusion were also analyzed.

**Results**: The averaged levels of bilirubin, ALT, AST, and ferritin were found to be high. Ferritin was positively correlated with ALT (r=0.508 and *p*<0.001) and AST ((r=0.569; p<0.001). There was no statistical difference in ferritin levels between the deferiprone and deferasirox groups ( *p*=0.776). However, higher total bilirubin and ALT were observed in the deferasirox group than in the deferiprone group ( *p*=0.001 and 0.022, respectively). Total ( *p*<0.001), indirect ( *p*<0.001), and direct bilirubin levels ( *p*=0.015) were significantly higher in patients with long-term transfusions than those receiving short-term transfusions. Higher ferritin was found with a statistical significance of *p*=0.008 in the long-term transfusions group.

**Conclusions**: Ferritin is high in people with transfusion-dependent β-thalassemia major and positively correlated with ALT and AST. Deferasirox might pose a higher risk of developing hepatic injury as compared with deferiprone. Yet, no significant change of deferasirox efficacy (based on ferritin level) was found between those receiving short-term and long-term transfusions.

## Introduction

Though the disease was known to be centered around the Southeast Asian and Middle Eastern regions, a recent review has highlighted the increasing prevalence of β-thalassemia in North America and Western Europe. ^1^ Approximately 1.5% of the world’s population are carriers of β-thalassemia, where the number varied across regions, including in specific countries. ^2^ For example, among Southeast Asian countries the number of β-thalassemia carriers ranged between 0.5% and 12.8% as reported in Myanmar and Malaysia, respectively. ^3^

People with β-thalassemia require a lifelong blood transfusion due to their inability to produce sufficient normal hemoglobin A. Ineffective erythropoiesis in β-thalassemia patients occurs because of the inherited genetic mutations causing impaired β-globin protein and consequently the imbalance in α- and β-globin chains production. ^4^ The condition leads to the inevitable hyperabsorption of irons deriving from either dietary irons or those from blood transfusion. ^5^ Excessive iron and its deposition could cause cardiac complications, which currently affects 25% of total 26,893 β-thalassemia major patients according to a meta-analysis. ^6^ To prevent the toxicity of iron overload, the patients should receive iron chelating therapy. The life expectancy of people with β-thalassemia major could be improved, even to be similar to those with β-thalassemia intermedia if managed with an iron chelating agent. ^7^

Based on a systematic literature review, a person with transfusion-dependent thalassemia should pay $128,062 for blood transfusion and iron chelating therapy. ^8^ In Indonesia itself, the access to iron chelation therapy is still a challenge, especially in rural areas. ^9^ It is worth mentioning that Indonesia lies in a thalassemia belt area owing to the high prevalence and carrier rate. ^10^ Therefore, it is of importance to focus the research on the Indonesian population to improve the clinical management and monitoring of iron overload among patients with β-thalassemia major. Herein, we compared two oral iron chelating drugs, deferiprone and deferasirox, prescribed to patients with β-thalassemia major and chronic transfusion. Deferiprone has two ligands which could coordinate with Fe forming a complex with three chelator molecules. ^11^ Meanwhile, deferasirox has three ligands that could coordinately bind one Fe atom to form a complex with two chelator molecules. ^11^ Understanding the performance of these two drugs in reducing the iron level could provide an insight into more effective and cost-efficient management.

In addition, we performed a correlation test between serum ferritin with serum alanine transaminase (ALT), aspartate transaminase (AST), and total bilirubin. The iron storage protein, ferritin, has been suggested as an indicator of iron overload and has been used to monitor iron chelation therapy efficacy. ^12^^,^^13^ Its correlation with liver enzymes could be an indicator for liver damage monitoring. ^14^^,^^15^

## Methods

### Study design

This research was conducted prospectively on children (1 to 18 years old) with β-thalassemia major who were hospitalized at the Children’s Thalassemia Center at Dr. Zainoel Abidin Hospital in Banda Aceh within the February–March 2022 period. The research aimed to seek the correlation between the administration of the iron chelating agent deferiprone with aspartate aminotransferase (AST), alanine aminotransferase (ALT), ferritin, and albumin. Ethical clearance was provided by the Ethical Committee of Dr. Zainoel Abidin Hospital, Banda Aceh (013/EA/FK-RSUDZA/2022, KEPPKN No 1171012P). Written informed consent for all the patients’ participation was obtained from the patients’ guardians.

### Patient recruitment

Diagnosis of β-thalassemia major was established based on hemoglobin (Hb) electrophoresis. The patient was included if having serum ferritin >1500 ng/mL, being treated with oral Ferriprox ^®^ or Exjade ^®^ (iron chelation therapies containing deferiprone and deferasirox, respectively), and they were not prescribed with other iron chelation therapy ( *i.e.,* injectable deferoxamine) within the last three months. Patients having histories of hepatitis virus (HPV) B and HPV C were excluded. Consecutive sampling was employed to select the study subjects with sampling size calculated based on Equation 1.

$$n=\frac{\left(\mathrm{Z\alpha}+\mathrm{Z\beta}\right)}{0.5\;\mathrm{Ln}\;\left(\frac{1+r}{1-r}\right)\;}+3$$

where,

α: Type I error at 5%

Zα: Standard alpha (1.64)

Β: Type II error at 10%

Zβ: Standard beta (1.28)

r: minimally significant correlation coefficient at 0.5.

### Data collection

Demographic characteristics data included in this study were sex, gender, and educational level, obtained by interviewing patients or their guardians. Nutritional status was calculated through anthropometric measurement from the patient’s body weight (kg) and height (m). The values obtained therein were categorized into obesity (>120%), overweight (>110–120%), healthy weight (90–110%), underweight (70–90%), and short stature (<70%).

### Serum parameters analysis

Venous blood (10 mL) was drawn from each subject and analyzed for serum bilirubin, ferritin, AST, and ALT. Serum ferritin level was obtained from an analysis using the immunoassay technique. Serum AST and ALT determinations were based on non-pyridoxal phosphate Inverse Fast Fourier Transform (IFFT).

### Statistical analysis

Continuous data were presented as mean±standard deviation (SD). Normality of the data distribution was judged based on a Kolmogorov-Smirnov test. Depending on the normality test, the significant difference was statistically analyzed using independent *t*-test or Mann-Whitney U test. All statistical analyses were performed on SPSS version 22 (SPSS Inc., Chicago, IL, USA) (SPSS, RRID: SCR_019096).

## Results

### Characteristics of patients

The patients included in this study were a balanced number of males (50%) and females (50%) with <10 years old as the most predominant age, followed by 10–15 years old and >15 years old, respectively ( Table 1). The average body weight and height of the patients were 25.56±9.75 kg and 125.96±18.06 m. As many as 45.1% of the total patients were in the healthy weight category, while another 45% were categorized as underweight. Almost all patients required 1 bag of blood each month. The average ferritin level of 3531.80±2322.44 ng/mL exceeded the normal range (7–140 ng/mL). The averaged total, indirect, and direct bilirubin levels were 1.86±1.00, 1.25±0.88, and 0.62±0.42 mg/dL, respectively. The averaged ALT and AST levels were also found to be higher than the upper limit for children (51.57±32.77 and 49.19±48.32, respectively). High SDs in ALT and AST indicate that there were some patients having these values in the normal range (10–40 UI/mL) but some were abnormal.

**Table 1.** Characteristics of the patients (n=102).

Variable	n (%)
Gender	
Male	51 (50)
Female	51 (50)
Age	
<10 years old	47 (46.1)
10-15 years old	41 (40.2)
>15 years old	14 (13.7)
Body weight, mean±SD (kg)	25.56±9.75
Height, mean±SD (m)	125.96±18.06
Nutritional status	
Short stature	7 (6.8)
Underweight	45 (44.1)
Healthy weight	46 (45.1)
Overweight	2 (2)
Obesity	2 (2)
Blood bag required per month	
>1 bag	3 (2.9)
1 bag	98 (96.1)
2 bags	1 (1)
Ferritin, mean±SD (ng/mL)	3531.80±2322.44
Total bilirubin, mean±SD (mg/dL)	1.86±1.00
Indirect bilirubin, mean±SD (mg/dL)	1.25±0.88
Direct bilirubin, mean±SD (mg/dL)	0.62±0.42
ALT, mean±SD (IU/L)	51.57±32.77
AST, mean±SD (IU/L)	49.19±48.32

### Correlations between serum ferritin and liver enzymes

Correlation tests were performed to see if the ferritin level was correlated with ALT, AST, or total bilirubin, where the results have been presented in Table 2. Based on Spearman’s rank correlation analysis, serum ferritin was found to be positively correlated with serum ATL based on r=0.508 and *p*<0.001. Similarly, serum ferritin was also positively correlated with serum AST with a statistical significance (r=0.569; *p*<0.001). These correlations suggest the increase in serum ferritin level contributes to the increase in serum ALT and AST levels. No correlation was found between serum ferritin and total bilirubin.

**Table 2.** Correlation between ferritin and ALT, AST, or total bilirubin (n=102).

Variable	R	*p*-value
Ferritin – ALT	0.508	<0.001 *
Ferritin – AST	0.569	<0.001 *
Ferritin – total bilirubin	-0.067	0.451

* Statistically significant at *p*<0.01 based on Spearman’s rank correlation.

### Comparisons between deferiprone and deferasirox

Comparisons of serum ferritin, bilirubin, ALT, and AST among patients receiving either deferiprone or deferasirox have been presented in Table 3. In this analysis, only 90 patients were included while 12 others were excluded because information about blood types was missing for some patients, or they had been treated with drug combinations. Levels of serum total, indirect, and direct bilirubin were found to be significantly different between the two groups ( *p*<0.05). Higher levels of serum ALT and AST were found in the deferasirox group as compared with the deferiprone group, yet statistical significance was only achieved in terms of serum ALT level.

**Table 3.** Comparisons between deferiprone and deferasirox based on levels of serum ferritin, bilirubin, ALT, and AST (n=90).

Variable	Deferiprone (n=50) Mean±SD	Deferasirox (n=40) Mean±SD	Statistic	*p*-value
Ferritin (ng/dL)	4237.45±3185.01	3941.82±2477.69	1075.000	0.776
Total bilirubin (mg/dL)	1.46±0.67	2.16±1.13	675.500	0.001 *
Indirect bilirubin (mg/dL)	0.93±0.50	1.50±1.04	681.500	0.001 *
Direct bilirubin (mg/dL)	0.53±0.34	0.65±0.44	822.000	0.029 *
ALT (IU/L)	47.66±29.23	60.14±34.71	807.500	0.022 *
AST (IU/L)	47.87±51.01	54.10±39.62	921.500	0.151

* Statistically significant at *p*<0.05 using Mann-Whitney U test.

We further analyzed the differences in serum ferritin, bilirubin, ALT, and AST levels between patients receiving long-term and short-term transfusions. The data of this analysis have been presented in Table 4. Significantly higher serum ferritin level was observed in the group that had undergone long-term transfusion ( *p*<0.05) among the deferiprone recipients. As in patients receiving deferasirox, long-term blood transfusion did not give a significant effect on ferritin levels. However, among those receiving deferasirox, long-term blood transfusion yielded significantly higher serum total, indirect, and direct bilirubin levels. Neither deferiprone nor deferasirox prescription yielded statistically different effects on serum ALT and AST levels between those undergoing long-term and short-term transfusions.

**Table 4.** Levels of serum ferritin, bilirubin, ALT, and AST in patients receiving long-term and short-term transfusions.

Variable	Long-term (n=21) Mean±SD	Short-term (n=29) Mean±SD	Statistic	*p*-value ^a^
Deferiprone (n=50)				
Ferritin (ng/dL)	4864.65±2957.53	3125.49±1394.99	-2.776	0.008 *
Total bilirubin (mg/dL)	1.52±0.64	1.3976±0.66	-0.630	0.532
Indirect bilirubin (mg/dL)	0.92±0.81	0.92±0.47	-0.009	0.993
Direct bilirubin (mg/dL)	0.59±0.42	0.48±0.28	259.000	0.371 ^b^
ALT (IU/L)	45.67±29.19	48.31±30.24	274.500	0.555 ^b^
AST (IU/L)	53.90±59.12	44.55±47.76	273.500	0.542 ^b^
Deferasirox (n=40)				
Ferritin (ng/dL)	4066.06±2959.85	4017.27±1941.73	183.50	0.668 ^b^
Total bilirubin (mg/dL)	2.72±1.24	1.53±0.60	66.50	<0.001 ^b^ *
Indirect bilirubin (mg/dL)	1.98±1.20	0.95±0.43	62.50	<0.001 ^b^ *
Direct bilirubin (mg/dL)	0.75±0.47	0.56±0.41	110.50	0.015 ^b^ *
ALT (IU/L)	65.76±37.45	56.74±32.34	161.00	0.307 ^b^
AST (IU/L)	56.95±55.00	53.89±47.79	169.00	0.421 ^b^

Transfusion category was divided into long-term (> 7 years) and short-term (≤ 7 years) based on the median.

^a^ Otherwise stated, *p*-value was obtained from independent t-test.

^b^ Obtained from Mann-Whitney U test.

* Statistically significant at *p*<0.05.

## Discussion

Increased ferritin levels in the sera of thalassemic patients are expected since they have ineffective erythropoiesis conditions, allowing hyperabsorption of dietary irons in the intestines. Multiple reports have suggested the effect of iron overload-associated liver abnormalities, indicated by the raised level of liver enzymes. Egyptian patients with β-thalassemia, aged 1.1 to 17 years old, had a mean serum ferritin level of 1367.52 ± 856.22 ng/mL, which exceeded the normal range in children (7–140 ng/mL). ^16^ Following the blood transfusion, irons are retained in the macrophages due to the increased hepcidin, hence elevation in serum ferritin. ^17^ Elevated ferritin as an iron storage marker was reported to have a strong correlation with iron overload condition. ^18^ Among 291 thalassemic children in Cipto Mangunkusumo Hospital, Indonesia, there was a predominance of mild, moderate, and severe degrees of iron overload (28.2%, 34.7%, and 27.8%, respectively). ^19^

Overaccumulation of iron in the liver could damage the organ following reactive oxygen species (ROS) formation through Haber-Weiss and Fenton reactions. ^20^ Ferric iron could be reduced to ferrous iron resulting in the formation of hydroxyl radicals which promotes DNA and protein damages, oxidation of amino acid side chains, and peroxidation of phospholipid. ^21^ In this present study, we found the abnormally high averaged values of serum AST and ALT (51.57±32.77 and 49.19±48.32, respectively), suggesting the presence of hepatic injury. The correlation tests revealed that serum AST and ALT levels are strongly influenced by ferritin, hence hepatoxicity of iron overload. Similarly, a study on 100 patients with β-thalassemia major reported the strong positive correlation between serum ferritin and AST or ALT. ^16^ Based on the study with a slightly higher number of transfusion-dependent thalassemic patients (n=138), ferritin was also found to be positively correlated with liver enzymes (AST and ALT). ^14^ Among patients with acute dengue fever, similar relationships among serum ferritin, AST, and ALT were also reported, where serum ferritin was proposed as an indicator for liver damage. ^15^ In the light of this iron toxicity, it is of importance for thalassemic patients undergoing blood transfusion to receive iron chelating therapies.

Herein, we observed differences of total, indirect, and direct bilirubin and ALT levels between deferiprone and deferasirox groups. All the aforementioned biomarkers were statistically higher in the latter group. In the correlation test, we did not find a correlation between ferritin and bilirubin, indicating that increased bilirubin was not associated with the efficacy of the iron chelator. In previous studies, hepatoxicity of deferasirox has been reported. ^22^^–^^24^ In drug-induced hepatic injury, the drugs induce the detrimental immune modulations after attaching to the enzyme molecules. ^25^ The neo-antigens eventually initiate the immune response cascade which could result in cytotoxicity and apoptosis. ^25^ Nonetheless, the reactions are delayed and required multiple exposures. Another proposed mechanism of the deferasirox-induced hepatic injury is the blockage of salt transport proteins (cholestatic injury), ^22^^,^^23^ which is supported by the findings of higher bilirubin levels in this present study. However, a case report suggested mitochondrial injury as the most explaining mechanism of deferasirox-induced liver injury which is consistent with elevations of transaminases. ^24^

The hepatoxicity effect of deferasirox was found to be more potent in patients with long-term transfusion as compared to those with short-term transfusion. However, interestingly, deferasirox could maintain its iron chelating efficacy even though the patients have undergone blood transfusion for more than 7 years. In the case of deferiprone, serum ferritin was produced at a higher level in patients with long-term blood transfusion. This is an indication that deferiprone lost its efficacy as an iron chelating agent as the patient received more blood transfusion. Our findings are in agreement with a study that continually monitored the long-term efficacy of deferasirox, which appeared to be sustained for 3.5 years. ^26^ Deferasirox uniquely has a longer half-life and bioavailability in the plasma (lasting for 24 hours) than other iron chelating therapies. ^27^ It is worth noting that, among patients receiving short-term blood transfusion herein, lower ferritin was observed in the deferiprone group than in the deferasirox group. In previous studies, deferasirox had a lower rate of effectiveness. ^12^^,^^28^

Our study had some limitations. For example, we did not simultaneously assess the effects of deferasirox or deferiprone in the thalassemic patients. The number of patients recruited in the study was too few, limiting the results in terms of being clinically meaningful. Moreover, we did not measure iron overload in specific organs, which could give an important insight since the drugs’ efficacies are dependent on the targeted organ. Diagnosis using magnetic resonance imaging (MRI) or other definitive tools should be carried out to conclude the hepatic injury in the patients.

## Conclusions

Patients with β-thalassemia major have a higher level of serum ferritin, which is strongly correlated with the elevations of serum ALT and AST levels, respectively. Deferasirox poses a higher risk of developing liver dysfunction than deferiprone, as suggested by increased levels of bilirubin and ALT. While deferiprone has reduced efficacy among patients managed with long-term blood transfusion, deferasirox could yield a similar efficacy regardless of the length of the chronic transfusion. These findings could be used as guidance for medical practitioners in determining the oral chelating therapies for iron-overload conditions among β-thalassemia major patients, by considering the net clinical benefits of each drug. Although a higher number of participants is required to obtain meaningful clinical conclusions, medical practitioners should be aware of the net clinical benefit of prescribing the oral chelators to transfusion-dependent β-thalassemia major patients.

## Data Availability

Figshare: ‘Comparison of oral iron chelators in the management of transfusion-dependent β-thalassemia major based on serum ferritin and liver enzymes’. DOI: https://doi.org/10.6084/m9.figshare.21564114. ^29^ This project contains the following underlying data:
‐ Master Data.xlsx. [Table containing the raw data of the study.] Data are available under the terms of the Creative Commons Attribution 4.0 International License (CC-BY 4.0).
