# Supplementary material for: Comparison of oral iron chelators in the management of transfusion-dependent β-thalassemia major based on serum ferritin and liver enzymes
Source: F1000Res. 2023 Dec 19;12:154. Originally published 2023 Feb 10. [Version 2] doi: 10.12688/f1000research.128810.2 (PMC11372351; doi:10.12688/f1000research.128810.2)
Supplement: Peer review history

# Reviewer report: Reviewer response for version 2

**DOI:** 10.5256/f1000research.160173.r231351

**Published:** 2024-09-03

**Authors:** Pustika Amalia Wahidiyat

**Affiliations:** 1 Department of Child Health, Division of Hematology-Oncology, Cipto Mangunkusumo General Hospital, University of Indonesia, Jakarta, Indonesia

No further comments for this article

Is the work clearly and accurately presented and does it cite the current literature?

Yes

If applicable, is the statistical analysis and its interpretation appropriate?

Yes

Are all the source data underlying the results available to ensure full reproducibility?

Yes

Is the study design appropriate and is the work technically sound?

Partly

Are the conclusions drawn adequately supported by the results?

Yes

Are sufficient details of methods and analysis provided to allow replication by others?

Partly

Reviewer Expertise:

Thalassemia

I confirm that I have read this submission and believe that I have an appropriate level of expertise to confirm that it is of an acceptable scientific standard.

# Reviewer report: Reviewer response for version 1

**DOI:** 10.5256/f1000research.141435.r208966

**Published:** 2023-11-24

**Authors:** Ahmed Yahya Dallal Bashi

**Affiliations:** 1 Al-Noor University College, Mosul, Iraq

Concerning the article titled *"Comparison of oral iron chelators in the management of transfusion-dependent* *β* *-thalassemia major based on serum ferritin and liver enzymes",* the work is a reasonable study to compare between 2 oral chelating drugs (Ferriprox (deferiprone) and Exjade (Deferasirox)) and their action in decreasing the level of serum ferritin and their effects on serum transaminases and serum bilirubin and the correlation between serum ferritin and the mentioned parameters. In my opinion the work was well done, and only few notes are required to be revised as follows:

1. Method: The doses of the 2 drugs were not mentioned which are very important as different protocols of treatment use different daily drugs doses.
2. Under the title " *serum parameters analysis*" methods for the estimation of serum; ferritin, AST, and ALT were mentioned while method of estimation of serum bilirubin was not mentioned.
3. Results: The mean length of the patients that was measured in meters was written as *"125.96* *±* *18.06".* This is in cm and if a meter unit is required then it is 1.2596±0.1806. The same fault was repeated in table 1.
4. Discussion: *"Following the blood transfusion, irons are retained in the macrophages due to the increased hepcidin, hence elevation in serum ferritin.17"*. Hepcidin production is suppressed due to erythropoietic activity which allows increased absorption of dietary iron in addition to the iron obtained from blood transfusion.
5. The authors used the scientific names of the drugs which are general names of the main constituent of the drug. I think they have to use the trade names of the drugs and in 2 brackets the constituents of the drugs as follows: Ferriprox (deferiprone) and Exjade (deferasirox).
6. Aim of the study was mentioned in the "background" only and have to be repeated in more details at the end of the "Introduction".

In addition, in aim of the study mentioned in the "Background" I suggest adding (on the liver) and substituting the word (toxicity) by (adverse effects) so it become (This study aimed to compare the performance and adverse effects of deferiprone and Deferasirox on the liver in β-thalassemia major patients).

1. In my opinion the authors went many times out of the scope of the aim of the study which leads to a relatively long article and can be summarized to fulfill the aim of the study.

Is the work clearly and accurately presented and does it cite the current literature?

Yes

If applicable, is the statistical analysis and its interpretation appropriate?

Yes

Are all the source data underlying the results available to ensure full reproducibility?

Yes

Is the study design appropriate and is the work technically sound?

Yes

Are the conclusions drawn adequately supported by the results?

Yes

Are sufficient details of methods and analysis provided to allow replication by others?

Yes

Reviewer Expertise:

Clinical Biochemistry with Special interest in Thalassemia

I confirm that I have read this submission and believe that I have an appropriate level of expertise to confirm that it is of an acceptable scientific standard.

# Response

**Published:** 2023-12-14

**Authors:** Sulaiman Yusuf

**Affiliations:** Universitas Syiah Kuala, Indonesia

Dear Reviewer,

 Thank you for your important suggestions. Here are our responses for each comments.

 1. Method: The doses of the 2 drugs were not mentioned which are very important as different protocols of treatment use different daily drugs doses.

**Response: The dosage of the iron chelation used in the study has been added in the article.**

 2. Under the title " *serum parameters analysis*" methods for the estimation of serum; ferritin, AST, and ALT were mentioned while method of estimation of serum bilirubin was not mentioned.

**Response: The method used to measure the serum bilirubin has been added under the heading of  "Serum parameters analysis". Thank you.**

 3. Results: The mean length of the patients that was measured in meters was written as *"125.96±18.06".* This is in cm and if a meter unit is required then it is 1.2596±0.1806. The same fault was repeated in table 1.

**Response: Thank you for pointing this issue. The values are now have been changed to metre: 1.25±0.18.**

 4. Discussion: *"Following the blood transfusion, irons are retained in the macrophages due to the increased hepcidin, hence elevation in serum ferritin.17"*. Hepcidin production is suppressed due to erythropoietic activity which allows increased absorption of dietary iron in addition to the iron obtained from blood transfusion.

**Response: The text have been revised. Thank you.**

 5. The authors used the scientific names of the drugs which are general names of the main constituent of the drug. I think they have to use the trade names of the drugs and in 2 brackets the constituents of the drugs as follows: Ferriprox (deferiprone) and Exjade (deferasirox).

**Response: Thank you for your suggestion. We have used the style “Ferriprox ® (iron chelation containing deferiprone) or Exjade ® (iron chelation containing deferasirox)” in Introduction (aim) and at the beginning of the Methods section. This is help authors to have detailed information of the drugs used. We avoid to write the name of trading names in all texts of the article since it might less appropriate. This might have perception that the study funded by the companies of the drug. In addition, we used one brand only for each of the drug in this study with is Ferriprox ® for deferiprone and Exjade ® for deferasirox.**

 6. Aim of the study was mentioned in the "background" only and have to be repeated in more details at the end of the "Introduction". In addition, in aim of the study mentioned in the "Background" I suggest adding (on the liver) and substituting the word (toxicity) by (adverse effects) so it become (This study aimed to compare the performance and adverse effects of deferiprone and Deferasirox on the liver in β-thalassemia major patients).

**Response: Thank you for raising this issue. The aim has been revised in the introduction and located at the end of the Introduction. It is now: “The objective of the study was to compare the performance and adverse effects of two oral iron chelating drugs (i.e., deferiprone (Ferriprox) and deferasirox (Exjade)) on the liver in β-thalassemia major patients with chronic transfusion. The effect on the liver were assessed by measuring the levels of serum ferritin, transaminase (ALT), aspartate transaminase (AST), total bilirubin, indirect bilirubin and direct bilirubin among patients with β-thalassemia major and chronic transfusion. In addition, we performed a correlation test between serum ferritin with ALT, AST, and total bilirubin.**

 7. In my opinion the authors went many times out of the scope of the aim of the study which leads to a relatively long article and can be summarized to fulfill the aim of the study.

**Response: Thank you for your suggestion. Since we have two main objectives: To compare the performance and adverse effects as well as to determine the correlation test between serum ferritin with ALT, AST, and total bilirubin; therefore, the article a quite long since we have to prepare the manuscript based on the aims of the study. Now the aims have been articulated clearly in the end of the Introduction.**

# Reviewer report: Reviewer response for version 1

**DOI:** 10.5256/f1000research.141435.r164926

**Published:** 2023-03-06

**Authors:** Pustika Amalia Wahidiyat

**Affiliations:** 1 Department of Child Health, Division of Hematology-Oncology, Cipto Mangunkusumo General Hospital, University of Indonesia, Jakarta, Indonesia

This study is a prospective study comparing two oral iron chelating drugs, deferiprone and deferasirox, in reducing iron overload and its effects on serum transaminase and bilirubin levels. This study also evaluated the correlation between serum ferritin with ALT, AST, and bilirubin.   

 There are some improvements needed for the manuscript:

 Introduction

1. Since similar studies with greater number of subjects have been established. Please provide the novelty of this study.
2. Please provide data from Indonesia

Methods

1. Please provide data about the dosage of iron chelation used in this study. 
2. Considering most of the patients with transfusion dependent thalassemia had organomegaly, please re-assessed the nutritional status based on the mid-upper arm circumference.
3. Since ferritin is easily affected by inflammatory status, please provide the technique to exclude the possibility of inflammatory state

Results

1. Please provide the analysis data between dosage used and the dependent variables
2. Please revised the height data in “metre” unit

Discussion

1. Please provide more discussion about the results findings, especially the difference between the two types of drugs.

Is the work clearly and accurately presented and does it cite the current literature?

Yes

If applicable, is the statistical analysis and its interpretation appropriate?

Yes

Are all the source data underlying the results available to ensure full reproducibility?

Yes

Is the study design appropriate and is the work technically sound?

Partly

Are the conclusions drawn adequately supported by the results?

Yes

Are sufficient details of methods and analysis provided to allow replication by others?

Partly

Reviewer Expertise:

Thalassemia

I confirm that I have read this submission and believe that I have an appropriate level of expertise to confirm that it is of an acceptable scientific standard, however I have significant reservations, as outlined above.

# Response

**Published:** 2023-12-14

**Authors:** Sulaiman Yusuf

**Affiliations:** Universitas Syiah Kuala, Indonesia

Dear Reviewer,

 Thank you for your important suggestions. Here are our responses for each comments.

**Introduction**

 1. Since similar studies with greater number of subjects have been established. Please provide the novelty of this study.

**Response: Thank you. We have added the novelty of the study. Mainly since most of the studies in Indonesia are from Java; studies outside of the Java is important to see of any differences.**

 2. Please provide data from Indonesia

**Response: The data of Thalassemia from Indonesia has been added in the text. The references have been adeed: Wahidiyat PA, Sari TT, Rahmartani LD, et al.: Thalassemia in Indonesia. Hemoglobin. 2022;46(1):39-44. doi: 10.1080/03630269.2021.2023565.**

Wahidiyat PA, Liauw F, Sekarsari D, et al.: Evaluation of cardiac and hepatic iron overload in thalassemia major patients with T2* magnetic resonance imaging. Hematology. 2017;22(8):501-507. doi: 10.1080/10245332.2017.1292614

**Wahidiyat PA, Wijaya E, Soedjatmiko S, et al.: Urinary iron excretion for evaluating iron chelation efficacy in children with thalassemia major. Blood Cells Mol Dis 2019;77:67-71. doi: 10.1016/j.bcmd.2019.03.007.**

**Methods**

 3. Please provide data about the dosage of iron chelation used in this study. 

**Response: The dosage of the iron chelation used in the study has been added in the article.**

 4. Considering most of the patients with transfusion dependent thalassemia had organomegaly, please re-assessed the nutritional status based on the mid-upper arm circumference.

**Response: Thank you for your suggestion; however, not all patients have the mid-upper arm circumference data in our database.**

 5. Since ferritin is easily affected by inflammatory status, please provide the technique to exclude the possibility of inflammatory state.

**Response: Thank you for your question. In all patients, the level of ferritin was measured only the patients had no infection (assessed through physically and laboratory tests including routine lab, C-reactive protein level and procalcitonin) and had no autoimmune diseases.**

**Results**

 6. Please provide the analysis data between dosage used and the dependent variables.

**Response: Thank you for your suggestion. Unfortunately, all the patients in our study were treated with the same dose of iron chelation drugs (deferiprone with dose 75 mg/kg body weight each day and deferasirox with dose of 20 mg/kg body weight each day for single dose).**

 7. Please revised the height data in “metre” unit

**Response: The unit of the height has been changed to unit metre.**

**Discussion**

 8. Please provide more discussion about the results findings, especially the difference between the two types of drugs.

**Response: More discussion has been added in particular the difference between the two types of oral chelating drugs (deferiprone and deferasirox) used in this study.**
